# The impact of gout as described by patients, using the lens of The International Classification of Functioning, Disability and Health (ICF): a qualitative study

**DOI:** 10.1186/s41927-020-00147-2

**Published:** 2020-08-12

**Authors:** Isobel Cairns, Karen Lindsay, Nicola Dalbeth, Cesar Díaz-Torné, Maria Antònia Pou, Basilio Rodríguez Diez, Enriqueta Pujol-Ribera, Charlotte Panter, Rob Arbuckle, Sophi Tatlock, William J. Taylor

**Affiliations:** 1 Rehabilitation Teaching and Research Unit, Department of Medicine, University of Otago, PO Box 7343, Wellington, New Zealand; 2 Department of Rheumatology, Auckland City Hospital, Auckland, New Zealand; 3 Department of Medicine, University of Auckland, Auckland, New Zealand; 4 Universitat Autònoma de Barcelona. Hospital de la Santa Creu i Sant Pau, Barcelona, Spain; 5 EAP Encants. Institut Català de la Salut, Barcelona, Spain; 6 Unitat de Reumatologia. Hospital Vall d’Hebró, Barcelona, Spain; 7 Institut Universitari d’Investigació en Atenció Primària, ICS, Barcelona, Spain; 8 Adelphi Values, Adelphi Mill, Bollington, Cheshire, SK10 5JB UK

**Keywords:** Gout, International classification of functioning, Disability and health, World Health Organisation, Patient outcome assessment, Qualitative research

## Abstract

**Background:**

The International Classification of Functioning, Disability and Health (ICF) aims to comprehensively describe the ways in which a person’s health condition affects their life. This study aimed to contribute to the development of an ICF core set for gout through patient opinion derived from focus groups and interviews.

**Methods:**

We conducted a secondary qualitative analysis of data from three studies investigating the patient experience of gout. In total there were 30 individual interviews and 2 focus groups (*N* = 17) comprising 47 participants. We conducted thematic analysis of the textual data to extract meaning units, which were then linked to the ICF.

**Results:**

A large number of ICF categories were relevant to patients with gout. Participants mentioned 93 third level categories, 17 of which were mentioned by more than 50% of patients. The most references for a single category was for *b280, Sensation of pain*, followed by *personal factors* (not yet categorised by the ICF). The most participants mentioned the environmental factor *e355, Health professional support*, followed by *b280, Sensation of pain*.

**Conclusion:**

The categories identified in this study as relevant to patients with gout highlight the severe pain associated with this disease, the impact on mobility and corresponding life areas. The roles of health professional support, medication, and personal attitudes to disease management are also reflected in the data. These results will contribute to the development of the ICF core set for gout.

## Background

Once known as the ‘rich man’s disease’, gout is increasingly common among older men, where it is the most common inflammatory joint disease [1]. Gout is a metabolic disease caused by high levels of urate, forming crystals that deposit in joints and periarticular tissues, leading to painful and debilitating arthritis [2]. Flares are intermittent but markedly painful, and as the disease progresses may become more frequent and tophi (deposits of monosodium uric acid crystals) may develop [2].

For the sufferer, gout attacks (or ‘flares’) are not only painful but disabling, leading to a marked decrease in mobility and impacting on many life areas, such as employment [3]. Yet previous means of assessing disability in gout have been problematic because of its episodic nature, and because measures may not adequately reflect the impact of gout on lower limb functioning and mobility or sufficiently capture the breadth of the disease impact as experienced by the patient [4, 5].

The International Classification of Functioning, Disability and Health is the World Health Organisation (WHO) framework for the conceptual understanding of health and disability [6]. It classifies the impacts of a disease related to its effect on body functioning, body structures or activities and participation, impacts which may be mediated by the facilitators or barriers offered by a person’s physical, social and cultural environment. The complete ICF contains over 1000 fourth level categories [6]. This provides a comprehensive overview of the health of an individual but only some of these will be relevant to a given disease.

The development of ICF core sets is intended to provide a concise set of categories of functioning related to a particular condition, leading to tools for research and practice that have global applicability [7]. Developing these measures requires input from both health professionals and sufferers of the disease. This paper supplements the research of Kool et al. by providing a patient perspective of the impact of gout on various areas of life as described by the ICF [8]. A core-set of ICF categories relevant to people with gout is highly useful to ensure adequate content coverage of tools and instruments to evaluate outcomes, including patient-reported outcomes, in clinical care, intervention studies and other outcomes research.

## Methods

This study was a secondary analysis of data collected by three primary qualitative studies, conducted in the United States [9], New Zealand [10] and Spain (not yet published). These studies are described in more detail below. There were 47 participants in total comprising 30 individual interviews and two focus groups with a total of 17 participants.

This study is a qualitative ‘amplified supplementary analysis’; more than one data set has been combined to allow an analysis of an aspect that was partially addressed in the primary studies – namely patient experience of gout – but that supplements these conclusions by including a new factor, the ICF [11]. Each individual study received approval from the relevant research ethics committee or Institutional Review Board.

### New Zealand transcripts

Ten individual interviews with male gout patients were obtained from a study based in South Auckland, NZ [10]. The authors used purposive sampling to ensure a range of ethnicities and experiences, conducting semi-structured interviews using a grounded theory methodology to explore participant experiences of gout, their understanding of the disease and attitude toward it and its treatment. Questions were motivated an overall question of “Why is gout so severe in Counties Manukau?” These included questions about the history of the patients’ lifetime experience of gout and pre-disease-onset knowledge of gout.

### United States transcripts

Transcripts were obtained from 20 individual interviews with gout patients (male = 12, female = 8) in Baltimore, New Orleans and St. Louis, USA [9]. Sampling was purposive to ensure a range of characteristics, and participants were recruited through their rheumatologist or primary care physician. The main motivation for this study was to understand the patient experience of gout in order to better measure clinical study endpoints or to guide development of a gout-specific outcome measure for clinical trial use. The interviews followed a detailed interview guide; the first half of the interview used open-ended questions to explore the participant’s experience of the disease with a main focus on symptoms and burden, and the second half involved completion and assessment of patient outcome measures, the Heath Assessment Questionnaire – Disability Index and Gout Assessment Questionnaire ‘overall concern’ domain. Each interview was on average 1.5 h long.

### Spanish transcripts

Two focus group transcripts were obtained from a study in Barcelona, Spain. The first focus group was purposively sampled to give a range of age, gender and disease characteristics and had 11 participants (M = 8, F = 3). Participants in the second focus group (*N* = 6; M = 5 F = 1) were recruited from an outpatient clinic and were selected consecutively. Each focus group lasted about 2 h and 30 min. Discussion prompts included: what causes gout, how gout was diagnosed, symptoms of gout, effects in daily life, experience of treatments, relationships with health professionals, and societal concepts of gout. Focus group discussion was in Castillian and translated into English by a multilingual, native Italian speaker.

### Analysis

Qualitative data analysis followed the process of meaning condensation [12]. Familiarity with the transcripts from each study was established and meaning units were identified in the data [12]. Concepts were identified within each meaning units and were grouped into similar concepts using the analysis software NVivo 11 (NVivo qualitative data analysis Software; QSR International Pty Ltd. Version 11, 2016). For instance, the comment, *“You can’t walk on it, you know. Even to have a – the sheet on the bed to touch it is painful”* contains the concepts ‘difficulty walking any distance’ and ‘pain caused from minimum sensation’. An initial sub-set of 2 transcripts was coded by both coders to establish interrater reliability.

Each concept was then linked to the most appropriate ICF category according to established linking rules [13, 14]. The ICF is divided into chapters, or ‘first level’ categories, which sub-divide the four separate concepts of body functions, body structures, activities and participation, and environmental factors. Each chapter consists of a number of ‘second level’ headings, which are made up of ‘third level’ and then the most detailed ‘fourth level’ categories (6). Concepts were linked to the most relevant fourth level ICF category if possible. If no such category was appropriate, they were linked to a third level category, or in some instances, a second level category. There were some concepts that were not able to be linked to any ICF categories, which were coded as non-definable.

As an example, the concept ‘difficulty walking any distance’ was linked to *d4508 Walking, other specified -- any distance* under the third level category, *Walking*. ‘Pain caused from minimum sensation’ was linked to *b2702 Sensitivity to pressure*, under the third level category, *Sensory functions related to temperature and other stimuli*. After concepts had been linked to the ICF the second researcher (WT) confirmed the accuracy of linkage decisions in a random subset (10%) of the linked categories.

## Results

In total 4897 meaning units were identified across all transcripts. These were grouped into 396 initial concepts. These concepts were linked to 93 third level categories of the ICF. Of these, 17 were in the body functions chapter, 5 in the body structures chapter, 35 in the activities and participation chapter, and 34 in the environmental factors chapter, 15 of which denoted facilitators or positive attributes of the environment, and the remaining 19 of which denoted negative attributes or barriers. See Table 1 for a comprehensive overview.

**Table 1:** Linked ICF categories and number of references by data source

ICF Category	US	NZ	Spain	Total
**Body functions**				
b body functions	4	8	8	20
b126 Temperament and personality functions	126	10	6	142
b130 Energy and drive functions	32	1	0	33
b134 Sleep functions	56	0	2	58
b140 Attention functions	7	1	0	8
b152 Emotional functions	117	11	3	131
b180 Experience of self and time functions	9	0	0	9
b265 Touch function	22	2	1	25
b270 Sensory functions related to temperature and other stimuli	81	12	7	100
b280 Sensation of pain	413	40	44	497
b298 Sensory functions and pain, other specified -- sensation of paralysis	16	0	0	16
b298 Sensory functions and pain, other specified -- sensation of weakness	10	0	0	10
b525 Defecation functions	7	5	1	13
b535 Sensations associated with the digestive system	2	3	0	5
b710 Mobility of joint functions	55	3	7	65
b755 Involuntary movement reaction functions	5	0	0	5
b770 Gait pattern functions	34	4	6	44
**Activity and participation**				
d activities	37	1	3	41
d230 Carrying out daily routine	28	1	0	29
d240 Handling stress and other psychological demands	5	2	3	10
d298 General tasks and demands, other specified -- planning to undertake activities	11	0	0	11
d4 mobility	63	5	8	76
d410 Changing basic body position	102	5	0	107
d415 Maintaining a body position	38	2	1	41
d420 Transferring oneself	28	3	0	31
d430 Lifting and carrying objects	10	0	0	10
d440 Fine hand use	35	1	0	36
d445 Hand and arm use	25	1	1	27
d450 Walking	148	16	8	172
d455 Moving around	33	3	2	38
d460 Moving around in different locations	24	5	4	33
d470 Using transportation	15	2	1	18
d480 Riding animals for transportation	0	1	0	1
d510 Washing oneself	27	1	2	30
d520 Caring for body parts	21	0	0	21
d530 Toileting	1	0	0	1
d540 Dressing	75	3	3	81
d550 Eating	8	0	0	8
d570 Looking after one’s health -- negative impacts	98	74	65	237
d570 Looking after one’s health -- positive impacts	105	59	28	192
d620 Acquisition of goods and services	33	0	0	33
d630 Preparing meals	15	0	0	15
d640 Doing housework	42	0	0	42
d660 Assisting others	10	0	0	10
d750 Informal social relationships	2	0	2	4
d760 Family relationships	7	8	5	20
d770 Intimate relationships	3	0	0	3
d845 Acquiring, keeping and terminating a job	4	3	1	8
d850 Remunerative employment	67	22	9	98
d870 Economic self-sufficiency	1	2	1	4
d920 Recreation and leisure	34	17	12	63
d930 Religion and spirituality	14	0	0	14
**Environmental factors**				
e110 Products or substances for personal consumption -- barrier	49	29	24	102
e110 Products or substances for personal consumption -- facilitator	170	42	32	244
e115 Products and technology for personal use in daily living	42	0	2	44
e120 Products and technology for personal indoor and outdoor mobility and transportation	64	3	3	70
e225 Climate	1	2	0	3
e310 Immediate family --	0	3	2	5
e310 Immediate family ++	61	26	8	95
e320 Friends --	0	1	0	1
e320 Friends ++	2	3	1	6
e325 Acquaintances, peers, colleagues, neighbours and community members ++	4	0	1	5
e330 People in positions of authority --	0	2	0	2
e330 People in positions of authority ++	2	6	0	8
e355 Health professionals --	3	30	23	56
e355 Health professionals ++	92	78	51	221
e355 Health professionals 00	2	3	5	10
e398 Support and relationships, other specified -- absence due to living alone	1	0	0	1
e398 Support and relationships, other specified -- burden of care for immediate family	1	6	0	7
e398 Support and relationships, other specified -- emotional impact for family members	3	3	1	7
e410 Individual attitudes of immediate family members --	0	3	1	4
e410 Individual attitudes of immediate family members ++	6	7	1	14
e420 Individual attitudes of friends ++	2	0	0	2
e420 Individual attitudes of friends 00	1	2	0	3
e425 Individual attitudes of acquaintances, peers, colleagues, neighbours and community members --	0	3	2	5
e430 Individual attitudes of people in positions of authority --	0	0	1	1
e430 Individual attitudes of people in positions of authority ++	3	2	0	5
e450 Individual attitudes of health professionals --	0	1	0	1
e450 Individual attitudes of health professionals ++	1	2	0	3
e460 Societal attitudes --	1	18	10	29
e460 Societal attitudes 00 or ++	1	4	0	5
e465 Social norms, practices and ideologies	10	15	2	27
e498 Attitudes, other specified -- restauranteurs	0	0	2	2
e570 Social security services, systems and policies ++	2	0	0	2
e580 Health services, systems and policies --	6	11	6	23
e580 Health services, systems and policies ++	2	8	8	18
**Body structures**				
s710 Structure of head and neck region	1	0	0	1
s730 Structure of upper extremity	27	3	2	32
s750 Structure of lower extremity	110	4	8	122
s798 Structures related to movement, other specified -- unspecified joints	26	2	1	29
s810 Structure of areas of skin	20	2	4	26
nd-ph -- tophi	1	4	11	16
personal factors	189	164	86	436
total	3071	829	542	4439

In addition, 27 initial concepts were linked to the concept of personal factors, which are not yet categorised by the ICF. These were grouped into four main areas: individual attitudes to disease and its management (60% of the references in this category), health literacy (19%), family experience of gout (12%) and negative attitudes to seeking care (8%).

There were several concepts were not definable by the ICF: co-morbidities, association with ethnicity, community visibility of gout, features of onset, physical (non-dietary) triggers, and certain approaches to managing pain and disease. Tophi were included as a non-definable physical factor.

There were 17 categories mentioned by at least 50% of participants; these categories largely correspond to those that received the greatest number of mentions (Table 2). The top five categories mentioned by the most number of participants were:

**Table 2:** Top 20 most mentioned ICF categories (ranked in order of most to fewest mentions)

ICF category	US (*n* = 20)		NZ (*N* = 10)		Spain (n = 17)	Total number of instances (% of participants)
	Number of instances (% of participants)	Median number of instances per participant	Number of instances (% of participants)	Median number of instances per participant	Number of instances (% of participants)	
b280 Sensation of pain	413 (100)	18.5	40 (100)	4	44 (88)	497 (96)
Personal factors	189 (100)	6.5	164 (100)	14	86 (82)	439 (94)
e110 Products or substances for personal consumption -- facilitator	170 (95)	7	42 (100)	4.5	32 (88)	244 (94)
d570 Looking after one’s health -- negative impacts	98 (95)	5	74 (100)	7	65 (94)	237 (96)
e355 Health professionals ++	92 (100)	5	78 (100)	8	51 (94)	221 (98)
d570 Looking after one’s health -- positive impacts	105 (85)	5	59 (100)	3.5	28 (76)	192 (85)
d450 Walking	148 (100)	7	16 (80)	1	8 (29)	172 (70)
b126 Temperament and personality functions	126 (95)	6.5	10 (60)	1	6 (41)	142 (68)
b152 Emotional functions	117 (85)	5.5	11 (80)	1	3 (12)	131 (57)
s750 Structure of lower extremity	110 (80)	5.5	4 (20)	0	8 (35)	122 (51)
^a^d410 Changing basic body position	102		5		0	107
e110 Products or substances for personal consumption -- barrier	49 (75)	2	29 (90)	3	24 (65)	102 (74)
b270 Sensory functions related to temperature and other stimuli	81 (65)	2.5	12 (60)	1	7 (35)	100 (53)
d850 Remunerative employment	67 (80)	2.5	22 (100)	2	9 (41)	98 (70)
e310 Immediate family ++	61 (65)	1	26 (70)	1	8 (41)	95 (57)
^a^d540 Dressing	75		3		3	81
d4 mobility	63 (80)	2	5 (30)	0	8 (29)	76 (51)
^a^e120 Products and technology for personal indoor and outdoor mobility and transportation	64		3		3	70
^a^b710 Mobility of joint functions	55		3		7	65
d920 Recreation and leisure	34 (70)	2	17 (60)	1.5	12 (41)	63 (57)

^a^were mentioned frequently by fewer than 50% of participants

e355 Health professionals ++ (98% of participants), eg *BM-02: In – one night, it was so severe, I had to go to the hospital, because I didn’t have any medicine. And I went on – went to XXXX Hospital. They gave me three tablets of Cortisine (*sic*) – Cortisine (*sic*) – what – how you pronounce it – and told me to go see my regular private-care physician, which was Dr. XXXX. And she gave me the same thing. And I’ve been taking it for the last 2 weeks, but I’m kind of over this bout a – at this time.*

d570 Looking after one’s health -- negative impacts (96%), eg *BM-03: It g – it got – it got a little better, but even though I don’t eat the beef, I don’t eat the steak, guess what? It just falls on you. Something just – just falls me on you, you know, one way or the other. You know, I think it’s just – it’s – it’s just falls, you know what I’m saying? Because –* b280 Sensation of pain (96%), eg *BM-04: Because that’s what overrides everything else. I mean the swelling is one thing. I mean you can kind of deal with that. The – the - uh, the temp – you know the heating up of the joint, you can kind of deal with that, but the excruciating pain is – you know, dominates all that.*

e110 Products or substances for personal consumption – facilitator, and personal factors. Eg *SUBJECT: Well he always tells me don’t eat this, don’t eat that, take these pills because they are the ones and he tried to put me on Allopurinol and they did not work because I was had an attack through it so his only alternative was to give me these pills Colchicine and of course it was down the track through other friends of mine that told me what Prednisone can do to you in the end and it will start eating into your bones or something like that and now I am starting to wake up to that. But at the end of the day it was fixing the pain and it was no fault of his, if I wanted some I was going to have to get some for the pain and all that.*

Categories from 17 of the 30 first level chapters of the ICF were mentioned by participants (Table 3). The Body Structures chapter was the least common, with only 5% of references coded to categories in this chapter; most of this related to Structures Related to Movement. The other chapters of Body Functions, Activities and Participation, and Environmental Factors were split roughly evenly, with 30, 39 and 26% of total references respectively (Table 4). In these first level categories, Sensory Functions and Pain was the most common in the category of Body Functions; the majority of this was related to b280 Sensation of pain (77% of the references for this chapter), followed by Temperament and personality functions (37%). Mobility and Self-Care were the most common in the category of Activities, with Walking the most common third-level category affected in Mobility (29%) and d570 Looking after one’s health -- negative impacts in Self-Care (42%). In the category of Environmental Factors, Products and Technology and Support and Relationships were the most common chapters, with e110 Products or substances for personal consumption – facilitator as the most common for the first (53%) and e355 Health professionals ++ as the most common for the second (52%).

**Table 3:** Most referenced ICF categories by percentage of participants

	number of participants				
Name	us N = 20	nz N = 10	spain N = 17	All	total as % of total participants
e355 Health professionals ++	20	10	16	46	98%
d570 Looking after one’s health -- negative impacts	19	10	16	45	96%
b280 Sensation of pain	20	10	15	45	96%
e110 Products or substances for personal consumption -- facilitator	19	10	15	44	94%
personal factors	20	10	14	44	94%
d570 Looking after one’s health -- positive impacts	17	10	13	40	85%
e110 Products or substances for personal consumption -- barrier	15	9	11	35	74%
d850 Remunerative employment	16	10	7	33	70%
d450 Walking	20	8	5	33	70%
b126 Temperament and personality functions	19	6	7	32	68%
e310 Immediate family ++	13	7	7	27	57%
d920 Recreation and leisure	14	6	7	27	57%
b152 Emotional functions	17	8	2	27	57%
b270 Sensory functions related to temperature and other stimuli	13	6	6	25	53%
b770 Gait pattern functions	17	3	5	25	53%
s750 Structure of lower extremity	16	2	6	24	51%
d4 mobility	16	3	5	24	51%
d455 Moving around	19	2	2	23	49%
e355 Health professionals --	3	7	12	22	47%
d activities	16	1	5	22	47%

**Table 4:** Distribution of affected ICF categories

	Number of instances				% of total references
	US	NZ	Spain	All	
b1 CHAPTER 1 MENTAL FUNCTIONS	347	23	11	381	10%
b2 CHAPTER 2 SENSORY FUNCTIONS AND PAIN	542	54	52	648	17%
b5 CHAPTER 5 FUNCTIONS OF THE DIGESTIVE, METABOLIC AND ENDOCRINE SYSTEMS	9	8	1	18	0%
b7 CHAPTER 7 NEUROMUSCULOSKELETAL AND MOVEMENT-RELATED FUNCTIONS	94	7	13	114	3%
**Total**				**1161**	**30%**
s7 CHAPTER 7 STRUCTURES RELATED TO MOVEMENT	164	9	11	184	5%
s8 CHAPTER 8 SKIN AND RELATED STRUCTURES	20	2	4	26	1%
**Total**				**210**	**5%**
d2 CHAPTER 2 GENERAL TASKS AND DEMANDS	44	3	3	50	1%
d4 CHAPTER 4 MOBILITY	521	44	25	590	15%
d5 CHAPTER 5 SELF-CARE	335	137	98	570	15%
d6 CHAPTER 6 DOMESTIC LIFE	100	0	0	100	3%
d7 CHAPTER 7 INTERPERSONAL INTERACTIONS AND RELATIONSHIPS	12	8	7	27	1%
d8 CHAPTER 8 MAJOR LIFE AREAS	72	27	11	110	3%
d9 CHAPTER 9 COMMUNITY, SOCIAL AND CIVIC LIFE	48	17	12	77	2%
**Total**				**1524**	**39%**
e1 CHAPTER 1 PRODUCTS AND TECHNOLOGY	325	74	61	460	12%
e3 CHAPTER 3 SUPPORT AND RELATIONSHIPS	171	161	92	424	11%
e4 CHAPTER 4 ATTITUDES	25	57	19	101	3%
e5 CHAPTER 5 SERVICES, SYSTEMS AND POLICIES	10	19	14	43	1%
**Total**				**1028**	**26%**

## Discussion

The ICF categories discussed by participants indicate that the most common bodily impacts of gout for patients are related to pain. Body structures most affected were swelling of areas related to movement. Given this, it is not surprising that the activity described most often as compromised was mobility, followed by self-care, but also including major life areas, particularly employment, domestic life and recreation. The impact of pain and limits on activity and mobility lead to impairments in mental and emotional function, as indicated by the number of references to temperament and emotions.

The frequency of references to categories related specifically to looking after one’s health under self-care reflects that the discussion of gout frequently touched on what triggered gout flares for participants and how they attempted to manage these triggers. For an individual what triggers a gout flare, such as particular foods, may not be obvious and thus their ability to make healthy choices is compromised. Also, avoiding enjoyed foods because they are triggers may be difficult for patients. The importance of factors related to self-management of the disease is also reflect in the frequency of meaning units coded under the (so far unspecified) ‘personal factors’ category of the ICF.

Environmental factors were an important feature for participants, both positive and negative – sometimes for the same category. For example, pharmaceutical treatments for gout were a facilitator for many participants but for others medication was ineffective or caused unpleasant side effects. Another factor was the support of health professionals, which could act as a facilitator when it was present, or a barrier when it was not – for example, lack of communication about the importance of managing the disease, or failure to refer on to more specialist care.

There was significant crossover between the frequency of ICF categories across study locations, but also some differences. It is a limitation of the study that such differences cannot be attributed to local culture but may have been influenced by the research protocol used in each location. Yet, the diversity of these patient groups and the large sample size is also one of the strengths of this study.

That this study was a secondary analysis of existing qualitative data is a limitation, as control could not be exercised over the collection of information to cover the ICF comprehensively with each participant. Different research objectives for each study meant that although there was a large quantity of data that was elicited in an unbiased manner, not all of it was relevant to the ICF. There are also issues combining studies with different methodologies – methodology is used to establish rigour and concepts such as ‘data saturation’, that indicate when the sample size is appropriate for the study. Because this concept was not under our control, the data was possibly ‘oversaturated’ – leading to excessive mentions of particular concepts while not reflecting the diversity of the experience of gout.

Nearly all the data coding was done by a single observer. This is acknowledged as a significant limitation, as the coding reliability cannot be calculated. However, it did mean that coding was consistent across the 3 data sources.

The concepts elicited from patients in these various studies cohere with previous investigations of the impact of gout on the life of patients, particularly the impact of affected lower limbs on mobility and other activities and taking time off work (3,4). Kool et al. found that outcome studies particularly failed to assess the ICF components of ‘Activity and participation’ and ‘Environmental factors’, both of which were found to be particularly relevant to participants in this study (8). The results of this study contribute to the process of developing an ICF Core Set for gout (7,8).

## Conclusions

In this multi-country qualitative study, we found that a large number of ICF categories are relevant to people with gout. Pain and personal factors are the most frequently mentioned ICF categories by people with gout. Environmental factors are also frequently relevant including dietary and other triggers, and the role of health professionals. The results of this study will contribute to the process of developing an ICF Core Set for gout.

According to the development process recommended by the ICF Research Branch, further research necessary to identify a core-set of ICF categories for gout are: (1) application of the generic ICF Checklist (https://www.who.int/classifications/icf/icfchecklist.pdf?ua=1) list of categories to patients with gout and (2) a consensus workshop of relevant stakeholders to consider all the data from preceding empirical research and to formulate the final core-set.

## Data Availability

The datasets generated and/or analysed during the current study are not publicly available due to the data consisting of confidential textual transcripts from individual participants.

## Abbreviations

ICF: The International Classification of Functioning, Disability and Health
NZ: New Zealand
USA: United States of America
WHO: World Health Organisation
